# Abnormal left-right organizer and laterality defects in *Xenopus* embryos after formin inhibitor SMIFH2 treatment

**DOI:** 10.1371/journal.pone.0275164

**Published:** 2022-11-07

**Authors:** Natalia Petri, Rhea Nordbrink, Nikoloz Tsikolia, Stanislav Kremnyov

**Affiliations:** 1 Department of Embryology, Faculty of Biology, Lomonosov Moscow State University, Moscow, Russia; 2 Anatomy and Embryology, University Medicine Göttingen, Göttingen, Germany; 3 Laboratory of Morphogenesis Evolution, Koltzov Institute of Developmental Biology RAS, Moscow, Russia; Imperial College London, UNITED KINGDOM

## Abstract

Left-right symmetry breaking in most studied vertebrates makes use of so-called leftward flow, a mechanism which was studied in detail especially in mouse and *Xenopus laevis* embryos and is based on rotation of monocilia on specialized epithelial surface designated as left-right organizer or laterality coordinator. However, it has been argued that prior to emergence of leftward flow an additional mechanism operates during early cleavage stages in *Xenopus* embryo which is based on cytoskeletal processes. Evidence in favour of this early mechanism was supported by left-right abnormalities after chemical inhibition of cytoskeletal protein formin. Here we analyzed temporal dimension of this effect in detail and found that reported abnormalities arise only after treatment at gastrula-neurula stages, i.e. just prior to and during the operation of left-right organizer. Moreover, molecular and morphological analysis of the left-right organizer reveals its abnormal development. Our results strongly indicate that left-right abnormalities reported after formin inhibition cannot serve as support of models based on early symmetry breaking event in *Xenopus* embryo.

## Introduction

Left-right symmetry breaking requires in most model vertebrates cilia-driven leftward fluid flow during neurulation and early somitogenesis stages [1]. Leftward flow is produced by rotating monocilia and has been shown to be located on the ventral surface of the gastrocoel roof plate (GRP) of amphibian, mammalian posterior notochord or related structure in zebrafish [2–5], areas designated as left-right organizer (LRO) [1]. Further studies indicate involvement of leftward flow in LR symmetry breaking of sea urchin [6]. In the next step the flow is transformed into asymmetric molecular processes mediated by immotile cilia located on lateral edges of LRO also known as somitic GRP in frogs [7] and crown cells in the mouse [8]. The immotile cilia are involved in sensing of leftward flow by PKD2 channel mediated calcium influx which in turn regulates at different levels *nodal* inhibitor *dand5 (coco)* [7, 9]. However absence of structurally equivalent ciliated left-right organizer in chick [10–12] and pig [12, 13] as well as in reptiles [14] does not support the universal role of the ciliary flow in vertebrates left-right symmetry breaking. Hence, alternative mechanisms at stages prior to the leftward flow also referred to as “early” were discussed as evolutionary conservative mechanisms [15–17]. Particularly serotonin and H^+^/K^+^-ATPase which are required for correct LR symmetry breaking in *Xenopus* have been proposed to break left-right symmetry by generating asymmetric ion flux at early stages [15, 18]. However, subsequent studies revealed that both H^+^/K^+^-ATPase subunit ATP4a and serotonin are required for correct ciliation suggesting involvement as upstream component in leftward flow [19, 20], although the role of serotonin is still a matter of contradiction [21, 22]. Alternatively, cytoskeleton based chiral processes were proposed to be basal and to operate already at the single cell level [23, 24]. The involvement of cytoskeleton has been supported by studies which have shown that chiral flow of myosin cytoskeleton is required in LR symmetry breaking in *C*. *elegans* [25] and that actin associated protein formin is critically involved in correct left-right patterning in snails [26, 27]. Moreover, formin perturbation achieved by small molecular compound SMIFH2 causes abnormalities of morphological left-right asymmetry also in *Xenopus laevis* hence indicating that conservative chirality-based mechanism of left-right symmetry breaking may operate at cleavage stages parallel to the flow based mechanism [27]. To clarify this issue, we investigated here the effect of formin inhibition in more detail, i.e. using the same inhibitor in different temporal contexts and analyzed how inhibition affects molecular left-right patterning, morphological laterality as well as the left-right organizer. Our data indicate that reported effect of chemical inhibition by SMIFH2 is caused by disturbed morphogenesis of the gastrocoel roof plate and disrupts thereby an upstream component of the flow based mechanism.

## Materials and methods

### Embryo culture and embryo explants

*Xenopus laevis* embryos were obtained by hormone-induced egg laying and *in vitro* fertilization using standard methods [28], de-jellied in 2% L-cysteine solution, pH 8, and then cultured in 0.1X MMR at 14 to 18°C. The embryos were staged according to the tables of normal development [29]. Before fixation the embryos were liberated from vitelline membrane with forceps. Dorsal explants were prepared at stage 18 after fixation as described by Shook and coworkers [30]. All protocols were developed in accordance with European convention for the protection of vertebral animals used for experimental and other scientific purposes (Strasbourg,1986) and approved by Ethic Committee for animal research of the Koltzov Institut of Developmental biology (approval number 53). Animals were anesthetized with MS222 for *in vitro* fertilization and tadpoles were anesthetized with MS222 prior to fixation.

### Formin modulation

*In vivo* modulation of formin functioning was performed with formin inhibitor SMIFH2 (Sigma, S4826) or formin agonist IMM-01 dissolved in 0.1x MMR with DMSO control. Embryos were treated with 1, 5, 10 μM and 50μM SMIFH2 concentrations or 10 and 100 μM IMM-01 concentrations. Experiments were performed on *X*. *laevis* embryos at developmental stages 2–6.5 (cleavage) or 10–18 (gastrula and neurula).

### Cloning and antisense RNA probes

*Nodal1*, *XlPitx2C* and *Sox17* cDNA were cloned using following primers:

*Nodal1*_forward: TTAATGCAAACCCTCCTTCTACCA
*Nodal1*_reverse: TCAAAACAACCTCATCTCCCTCAT
*XlPitx2C*_forward: GCTGCAGATGGAACCGAAGGAA
*XlPitx2C*_reverse: GGGAGGTGTTGGGGGAGCATAA
*Sox17*_forward: TGGCAAGTCGTGGAAGTCTC
*Sox17*_reverse: CTATAGGGCACGGTGTCAGTCT

Amplified fragments were cloned into the pAL2 vector (Evrogen, Russia).

PCR products from the plasmids were amplified with T7 and Sp6oligos and used for generation of digoxigenin-labeled RNA (Roche) antisense probes.

*Dand5 and Tekt2* PCR products for probe synthesis were amplified from previously published plasmids [9, 31].

### *In situ* RNA hybridization

Wild-type or formin-modulated embryos were fixed in 4% formaldehyde in PBS for 2 hours, stored in 100% ethanol at -20°C. Whole-mount embryos or dorsal explants were processed following modified published protocol [32]. All steps were performed in glass vials. Briefly: fixed samples were rehydrated in 75% ethanol, 50% ethanol and 25% ethanol in PBS with 0.1% Tween-20 (PTw) for 5 minutes each, washed three times in PTw for 5 minutes each, then incubated in 10 μg/ml proteinase K in PTw for 3.5 (dorsal explants) to 17 (tailbud stages) minutes. Digestion was stopped by washing twice in freshly prepared triethanolamine solution (TEA), then in 0.25% and 0.5% acetic anhydride in TEA for 5 minutes each. Samples were washed twice in PTw for 5 minutes each and re-fixed in 4% paraformaldehyde in PTw for 20 minutes at room temperature. Samples were washed four times in PTw for 5 minutes each, in 20% hybridization buffer (HB) in PTw and 100% HB for 10 minutes each and prehybridized in HB at 60°C for 12 h. Samples were hybridized at 60°C overnight in probe solution (HB containing 300 ng/ml of antisense RNA probe). Samples were washed in prehybridization solution for 1 hour at 60°C, then three times in 2x SSC (pH 7) at 60°C for 20 minutes each, twice in 0.2x SSC (pH 7.0) at 60°C for 30 minutes each and finally twice in maleic acid buffer (MAB; 0.1M maleic acid, 0.15M NaCl (pH 7.5)) at room temperature for 15 minutes each. Samples were treated with blocking solution (1% blocking reagent Roche/MAB) for 2,5 h at room temperature and then with 0.1% anti-digoxigenin AP antibodies in blocking solution overnight at 4°C. Samples were washed ten times for 1 h in MAB at room temperature and stained in BM purple substrate (Boehringer) at 4°C in the dark (a few hours to several days). After staining, samples were fixed in 4% formaldehyde/PBS for 2 hours, bleached in a solution of 0.5x SSC, 5% formamide and 5% H_2_O_2_ for 1 to 4 h under bright light to remove pigmentation (for stage 28 embryos), re-fixed in 4% formaldehyde/PBS for 2 hours, transferred to ethanol and then to glycerine for long-term storage at -20°C. Samples were examined at Olympus SZX9 stereomicroscope (Olympus, Japan).

### Immunofluorescent analysis of GRP

Wild-type or formin-modulated embryos at stage 18 were fixed in a solution of 4% formaldehyde in PBS overnight at 4°C, dorsal explants were prepared. Samples were washed in PBS for 30 minutes, in PTw for 1 h, then in PTw with 20% heat-inactivated goat serum for 2 h, and stained with anti-acetylated tubulin primary mouse antibodies (1:1000 in PTw with 20% serum) overnight at 4°C. After primary staining samples were washed five times in PBS for 1 h each, in PTw with 20% serum for 2 h, and incubated with secondary anti-mouse antibodies (1:1000 in PTw with 20% serum) overnight at 4°C. Samples were then washed three times in PBS for 15 minutes each, in PTw for 1 h, and stained with 5ng/ml TRITC-Phalloidin in PTw for 45 minutes at room temperature in the dark, then washes twice in PBS for 1 h each and transferred to 80% glycerine in PBS for long-term storage at -20°C.

Confocal microscopy of stained samples (focused to the gastrocoel roof plate) was performed on confocal laser-scanning microscope Olympus FluoView FV10i (Olympus, Japan).

### Light microscopy

For histological analysis, dorsal explants were fixed in Bouin solution, dehydrated through an ethanol series and 100% acetone, embedded in epoxy resin and sectioned on an ultratome (Tesla, Czech Republic). 2–3 μm sections were stained by 1% toluidine blue and examined with a Axio Imager A1 (Carl Zeiss) microscope.

### Scanning electron microscopy

Wild-type or formin-modulated embryos at stage 18 were fixed in 2.5% glutaraldehyde in 0.1M cacodylic buffer overnight, dorsal explants were prepared. Samples were postfixed in OsO_4_, dehydrated through an ethanol series and 100% acetone, critical point dried. Dried specimens were mounted on taps with conducting silver and sputtered with Gold-Palladium. Samples were examined and photographed with a CamScan S-2 microscope.

### Morphometric measurements and data analysis

Morphometric measurements were made with the open-souce program ImageJ version 1.37 (http://rsb.info.nih.gov./ij/). Statistical significance was determined using the R program (R Development Core Team, 2004).

We determined cilia parameters of GRP cells as described in [20]. A square at the center of the GRP was selected in SEM pictures for manual analysis of cilia length and polarization (posterior, central, other). The ciliation rate was calculated as the ratio of cilia over cells (separately in each GRP SEM photograph). Quantitative data from ImageJ measurements were analyzed with pairwise t test with Bonferroni correction.

To analyze *nodal1* and *pitx2* expression in stage 28 embryos and heart and gut asymmetry in tadpoles we divided the observed phenotypes into groups and counted the number of samples in each group. Statistical calculations of gene expression patterns and visceral organs asymmetry of formin-modulated embryos were performed with two-proportions Z-test with Bonferroni correction.

## Results

### Formin inhibition at gastrula-neurula stages disturbs molecular laterality and organ situs

**Fig 1:**
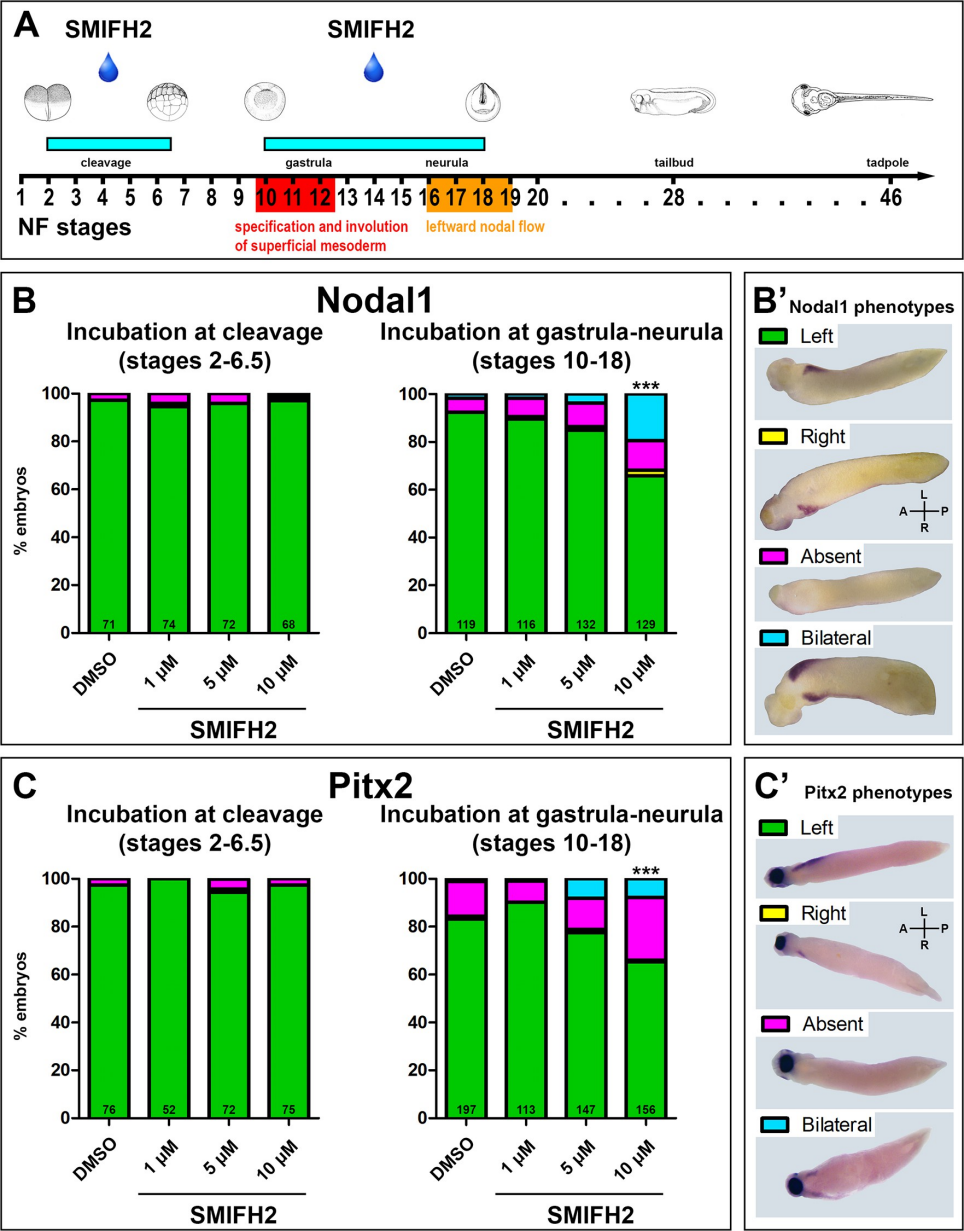
Molecular left-right patterning after formin inhibition. (A) Scheme of time course of chemical treatment and stages of LR analysis. (B, B’) Formin inhibitor SMIFH2 had no effect on *nodal1* expression patterns in the embryos treated during cleavage (N = 3) and reduced the proportion of left-sided *nodal1* expression in the embryos treated during gastrulation and neurulation (N = 6, p = 0,00000313 for 10μM SMIFH2). (B) Summary of experimental data. (B’) Representative images of formin-modulated stage 28 tailbud embryos displaying left-sided and abnormal (right, absent and bilateral) expression of *nodal1*, ventral view. (C, C’) Formin inhibitor SMIFH2 had no effect on *pitx2* expression patterns in the embryos treated during cleavage (N = 4) and reduced the proportion of left-sided *pitx2* expression in the embryos treated during gastrulation and neurulation (N = 7, p = 0,0005445 for 10μM SMIFH2). (C) Summary of experimental data. (C’) Representative images of formin-modulated stage 28 tailbud embryos displaying left-sided and abnormal (right, absent and bilateral) expression of *pitx2*, ventral view. A, anterior; L, left; P, posterior; R, right. ***, p-value<0.001 compared with the DMSO control, two-proportions z-test with Bonferroni correction; N, number of experiments. Numbers at the base of columns represent number of analyzed embryos.

Considering controversial data concerning lethal dosage [27, 33, 34] we first tested the optimal concentrations of the formin inhibitor SMIFH2. Embryos were treated either during cleavage or during gastrula-neurula stages and the proportion of embryos survived to the tailbud stage (NF stage 28) was used as a read out. SMIFH2 at a 50 μM concentration reveals a general toxicity regardless of treatment stage and resulted in 100% lethality (S1A and S1B Fig). In order to define the developmental period when formins are required for laterality establishment, we treated embryos at stages 2–6,5 NF (during cleavage) and at stages 10–18 (gastrula-neurula) (Fig 1A). The embryos were then analyzed for *nodal1* and *pitx2* expression patterns at stage 28 (Fig 1B and 1C).

We subdivided detected *nodal1* expression patterns into 4 types: the expression at the left side which correlates with normal laterality, as well as right-sided, bilateral or absent expression (Fig 1B’).

The embryos treated with SMIFH2 during cleavage showed no significant change of *nodal1* expression, whereas SMIFH2 administration at gastrula-neurula stages led to a dose-dependent decline of the proportion of embryos with left-asymmetric *nodal1* expression pattern which was significant for 10μM concentration of inhibitor (Fig 1B).

Similarly, to *nodal1*, we observed four expression patterns of *pitx2* (left-asymmetric, right-asymmetric, bilaterally symmetric and absent). Again, incubation of *Xenopus* embryos with formin inhibitor SMIFH2 at cleavage stages did not significantly affect *pitx2* expression, and formin inhibition in embryos at gastrula-neurula resulted in significantly lower proportion of left-asymmetric patterned embryos for higher dose of inhibitor (Fig 1C). At stage 28, we did not observe any specific features of phenotype in treated embryos.

**Fig 2:**
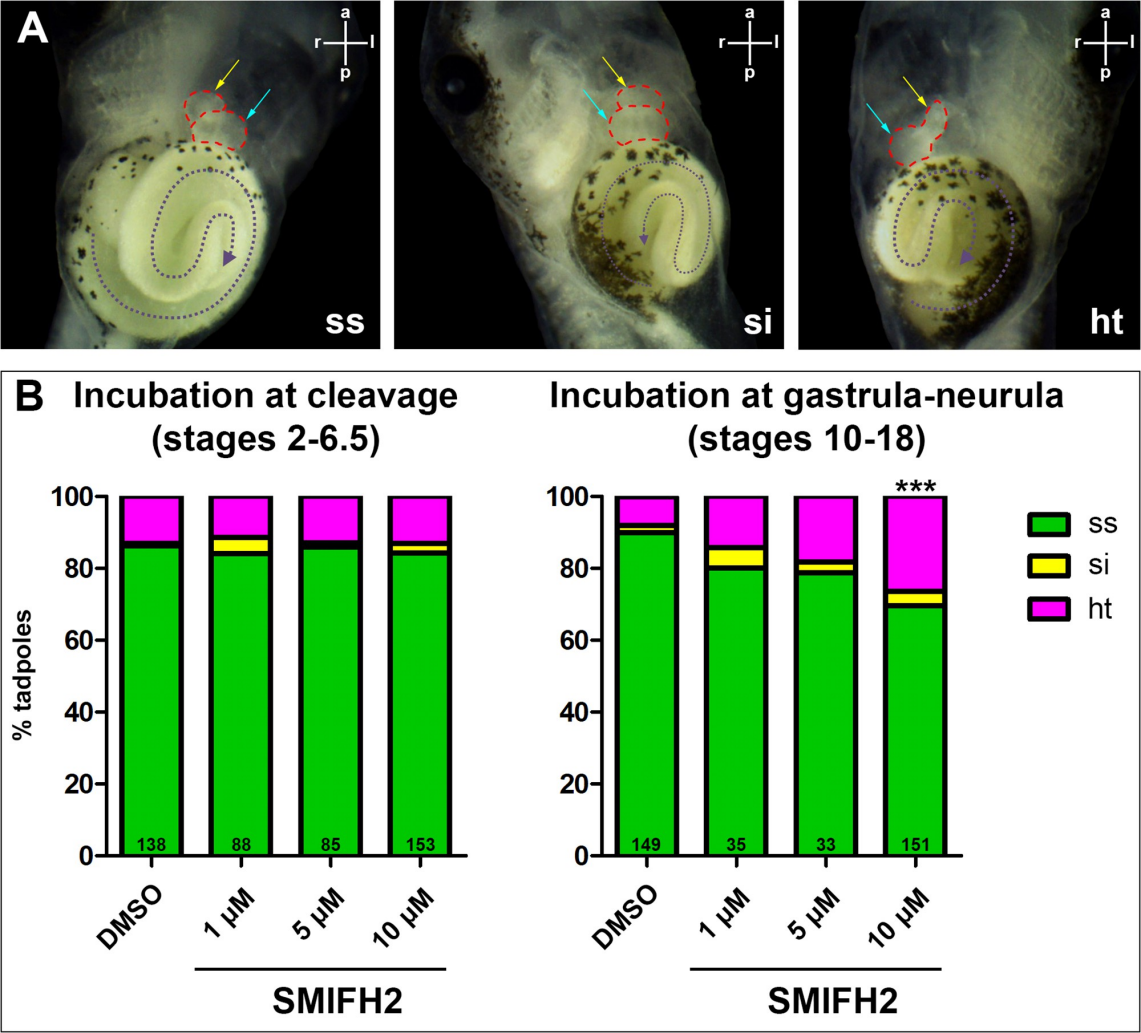
Visceral organ situs after formin inhibition. (A) Representative images of formin-modulated stage 46 tadpoles displaying normal organ situs (ss), situs inversion (si), or heterotaxia (ht), as determined by the direction of heart looping (outlined by dashed red line) and gut coiling (outlined by dashed violet line), ventral view. (B) Formin inhibitor SMIFH2 had no effect on organ situs in the tadpoles treated during cleavage (N = 5) and reduced the proportion of tadpoles with normal organ laterality after treatment with 10 μM SMIFH2 during gastrulation and neurulation (N = 5). a, anterior; ht, heterotaxia; l, left; p, posterior; r, right; si, situs inversus; ss, situs solitus. Cyan arrows indicate heart ventricle, yellow arrows indicate truncus arteriosus. ***, p-value = 0,00006552 compared with the DMSO control, two-proportions z-test with Bonferroni correction; N, number of experiments. Numbers at the base of columns represent number of analyzed embryos.

Since asymmetric molecular patterning is followed by morphological asymmetry in the course of development, we wondered whether the observed effects of formin inhibition would persist in organ laterality of tadpoles and repeated formin inhibition in embryos during cleavage or gastrula+neurula stages, now allowing them to grow up to stage 46. The phenotypes of tadpoles were analysed for heart and gut laterality and classified into *situs solitus*, *situs inversus* and heterotaxy (Fig 2A). The analysis of laterality of tadpoles which underwent incubation in formin inhibitor during cleavage does not reveal differences from the control group, whereas the tadpoles exposed to inhibitor at gastrula-neurula stages showed a significantly lower proportion of embryos with *situs solitus* after a higher dose of inhibitor (Fig 2B).

### Formin inhibition perturbs GRP region

**Fig 3:**
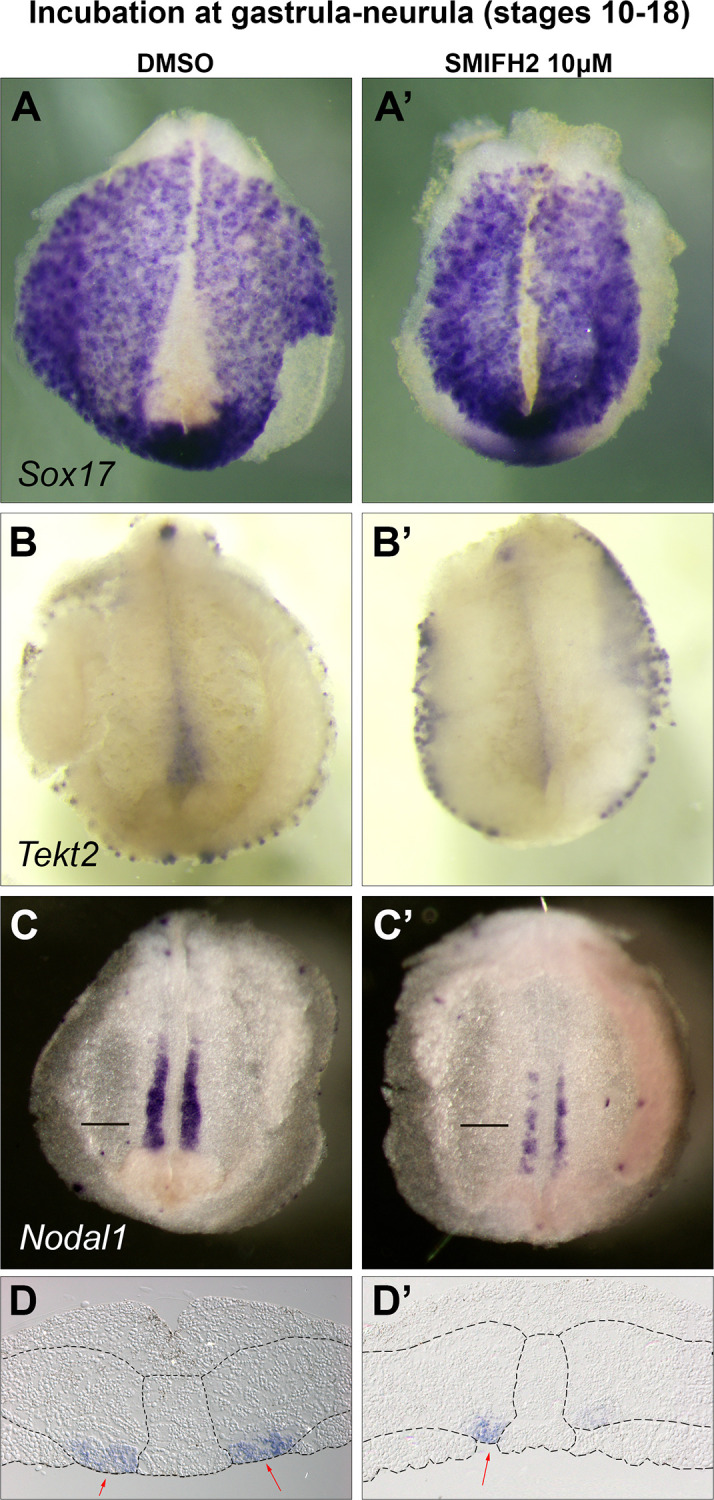
Formin inhibition at gastrula and neurula stages results in abnormal molecular patterning of GRP. (A-B’) Representative images of molecular patterning in the GRP. Dorsal explants of stage 18 neurula embryos after control treatment (A,B) and SMIFH2 administration (A’,B’) at stages 10–18. *In situ* hybridisation was performed with probes specific for *sox17* (A,A’) and *tekt2* (B,B’). (C and C’) Representative images of *nodal1* expression pattern in the GRP. Dorsal explants of stage 18 neurula embryos after control or SMIFH2 treatment at stages 10–18. (D and D’) Histological sections through GRP demonstrate the position of *nodal1-*positive cells in the prospective somitic mesoderm. Arrows indicate areas of superficial *nodal1*-positive cells.

As the effect of formin inhibitor is revealed after treatment at the stages of gastrula and neurula, we focused our attention on the so-called left-right organizer which emerges in the GRP at this time. First we studied whether the modulation of the formin activity influences the molecular anatomy of the left-right organizer and analyzed expression of endodermal marker *sox17* [35] and of medial GRP marker *tekt2* [31] after SMIFH2 treatment. Analysis of *sox17* expression in control embryos reveals negative area of characteristic triangular shape at the posterior dorsal midline of embryo, corresponding to the zone of LRO at this time of development (Fig 3A, S2C Fig). In inhibitor-treated embryos this negative area displays slit-like shape indicating narrowing of surface of LRO (Fig 3A’, S2C Fig). Expression of *tekt2* reveals a complementary pattern with triangular LRO-related domain in control embryos and slit-like domain in treated embryos (Fig 3B and 3B’, S2D Fig). Taking together treated embryos reveal excessive covering of GRP with *sox17*-positive cells which is accompanied by narrowing of its medial area expressing marker of cilia *tekt2*. Complementary pattern of *tekt2* and *sox17* expression is already seen at late gastrula stages while the expression pattern of both markers in SMIFH2 treated embryos is at this stage similar to the DMSO control (S2A and S2B Fig).

Next we analyzed markers of lateral, somitic GRP. The embryos were treated with 10 μM concentration of formin inhibitor during cleavage or at the stages of gastrula-neurula, as described before, and then analyzed for *nodal1* expression at the stage 18. *nodal1* expression was seen both in SMIFH2 treated and control embryos while embryos treated at gastrula-neurula stages display weaker expression (Fig 3C and 3C’, S3A Fig). Similarly, both treated and control embryos express *nodal* antagonist *dand5* (*coco*) (S3B Fig). Transverse technovit histological sections demonstrate that *nodal1* is expressed in the ventral part of parachordal mesodermal domain in both control embryos and embryos treated with high concentrations of inhibitor. However, while in control embryos the *nodal1*-positive cells form a superficial domain located in the gastrocoel roof, the *nodal1*-positive cells of inhibitor-treated embryos are located mainly in the indentation partly covered by endodermal cell layer (Fig 3D’ and S4 Fig). Sections of *dand5* support deep localization of somitic GRP (S4 Fig).

**Fig 4:**
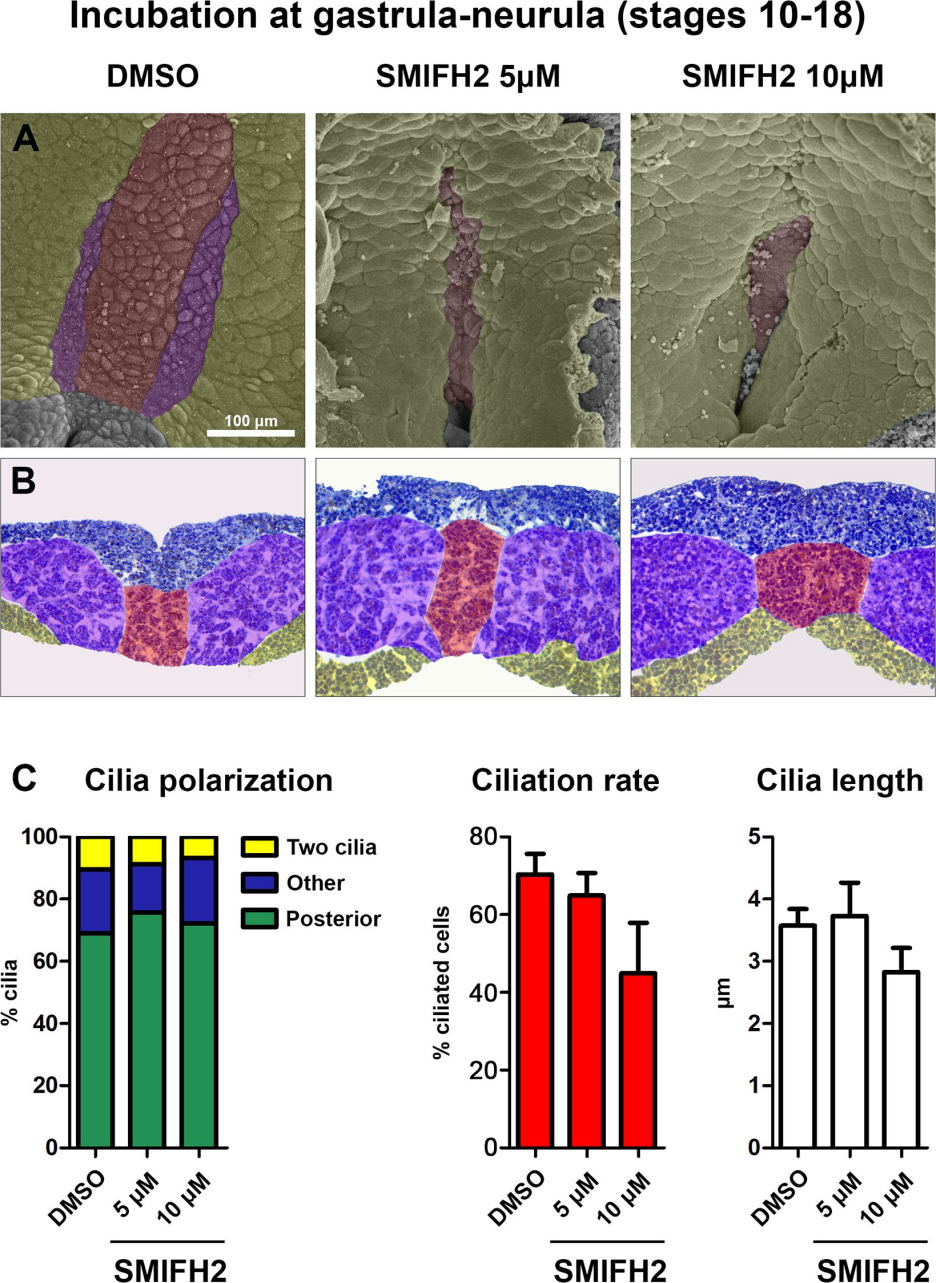
Formin inhibition at gastrula and neurula stages results in morphology defects of left-right organizer at stage 18. (A) Representative scanning electron microscopy images of GRP after exposure to various concentrations of formin inhibitor. (B) Representative histological sections through the GRP after exposure to various concentrations of formin inhibitor. Blue color indicates ectoderm, red–notochord and hypochord, violet–somitic mesoderm, yellow–endoderm. (C) Cilia polarization, cilia length and percentage of ciliated cells (ciliation rate) in embryos treated with DMSO and SMIFH2 (5 μM and 10 μM). Measurements were performed in notochordal GRP of 11, 14 and 9 embryos respectively (number of analysed cells = 303, 453 and 234 respectively). The apparent decrease of number of ciliated cells is non-significant (p-value = 0,295 for 10 μM, t-test with Bonferroni correction).

In the next step, we examined the morphology of the GRP (Fig 4A–4C) in treated and control embryos at stage 18, when all morphological characteristics of the ciliary organizer reach their maximum. SMIFH2 treated embryos reveal at stage 18 phenotypic peculiarity displaying a brick-like shape while the embryos do not fill the space bounded by vitelline membrane.

SEM images of GRP in *Xenopus* embryos demonstrate that in comparison to control group the central area of GRP in treated embryos is notably narrower revealing slit-like shape and its lateral sides are covered by larger cells (Fig 4A).

This arrangement can be visualized by immunofluorescent analysis of GRP which reveal a central area covered by small cells bearing long mostly polarized monocilia (S5 Fig) and transverse histological sections through GRP which suggest that the lateral area of LRO in treated embryos is partially covered by endodermal cells (Fig 4B). Importantly, SMIFH2 treatment does not affect cortical actin (S5 Fig). Since the medial areas of the GRP were only incompletely covered by endoderm we were able to study ciliation features: the percentage of ciliated cells at the dorsal midline (measured in the uncovered area) insignificantly declined in treated embryos, and the polarization of ciliated cells judged on the posterior position of cilia did not change, neither did the cilia lengths (Fig 4C and S6 Fig).

Finally, we analyzed morphology and expression pattern of GRP-related genes in embryos treated at cleavage stages and found no signs of endodermal covering of left-right organizer at stage 18 (S7 Fig).

### Left-right perturbation after treatment with formin agonist IMM-01

To address specificity of SMIFH2 effect we treated embryos at cleavage or at gastrula-neurula stages with formin agonist IMM-01 and analyzed *nodal1* and *pitx2* expression at stage 28 as well as the morphology at stage 18. Whereas administration of 10 μM of IMM-01 at cleavage stage was not followed by significant changes of *nodal1* or *pitx2* expression, treatment with 100 μM led to significant decrease of (left-sided) asymmetry of *nodal1* expression. In embryos treated at gastrula-neurula stages treatment with 100 μM led to significant decrease of both *nodal1* and *pitx2* asymmetry (S8A and S8B Fig). We examined the morphology of the GRP in embryos at stage 18 treated with IMM-01 at gastrula-neurula stages. The morphology of the left-right organizer does not reveal endodermal covering of GRP (S8D–S8F Fig) which is seen in embryos treated with SMIFH2 at gastrula-neurula stages. The percentage of ciliated cells at the dorsal midline insignificantly declined in treated embryos, and the polarization of ciliated cells judged on the posterior position of cilia did not change. The average cilia length, however, declined significantly in IMM-01 treated embryos compared to DMSO control (S8C Fig).

## Discussion

Our study aimed to analyze the temporal dimension and morphological details of previously reported [27] effect of chemical formin inhibition on left-right symmetry breaking and patterning in *Xenopus*. We found that SMIFH2 administration perturbed morphological laterality of visceral organs, the molecular patterning studied by expression of *nodal1* and *pitx2* in the lateral plate mesoderm as well as the structure of the left-right organizer. Hence, presented data supports and extends reported effect of SMIFH2 treatment on left-right symmetry breaking in *Xenopus*. Though, our analysis reveals that the significant effect of the SMIFH2 administration was observed only after treatment at gastrula-neurula stages and is dose-dependent. Furthermore, our data is consistent with early findings [33, 34], suggesting that SMIFH2 in concentrations 50μM used in [27] is lethal to embryos regardless to development stage, a difference which may be explained by intracellular SMIFH2 breakdown or inactivation [34].

Results of our investigation suggest that reported abnormal development of situs in *Xenopus* after SMIFH2 administration cannot be used in favour of proposed LR symmetry breaking during cleavage stages. The molecular mechanism of observed effect remains open since SMIFH2 has been shown additionally to modulate myosin retrograde flow and contractility [36]. The specific role of formin in left-right symmetry breaking is supported by situs abnormalities after gain of function experiment performed by microinjection of mouse Dia1 formin into *Xenopus* animal pole and in dorsal left or ventral right blastomeres [27]. However, reported experimental setting does not allow to study the temporal dimension of effect and cannot be used to discriminate between early and late hypothesis. Importantly, descendants of ventral blastomeres are involved in morphogenesis of the posterior archenteron roof and somite formation and may therefore disturb the function of the left-right organizer [37–39]. Treatment of *Xenopus* embryos with formin agonist IMM-01 led to significantly abnormal *nodal1* pattern in the lateral plate mesoderm irrespective of the stage of treatment and therefore does not allow to discriminate between early versus late effect. However, our data indicates that effect of IMM-01 treatment is not due to endodermal covering of GRP and requires an alternative mechanism interfering with left-right patterning. SMIFH2 and IMM-01 target different parts of formin protein: while SMIFH2 binds to FH2 domain of formin molecule [33], IMM-01 disrupts the binding of DID-DAD domains and prevents the formin autoinhibition [40]. Whether observed shortening of cilia disturbs the leftward flow and thereby provides a possible mechanism of effect of IMM-01 remains to be clarified.

Our analysis of morphology and expression pattern of GRP after SMIFH2 treatment revealed extended lateral to medial covering of the left-right organizer surface by endoderm. The extent of the covering varies and the observed effect can be explained by both disturbed flow and disturbed flow sensing. Most of SMIFH2-treated embryos still keep open medial GRP area which bears correctly polarized monocilia. Hence, taking into account that the ciliary organizer with radically reduced number of cilia is still able to produce effective leftward flow [41] a most parsimonious explanation would be a disabled sensing of the leftward flow and further propagation of left-right signaling. Reduction or loss of somitic GRP has been reported in embryos with inhibited FGF signaling [42]. However, this observation was accompanied by disappearance of *nodal1* expression domain while in SMIFH2 treated embryos *nodal1* domain was suppressed but still detectable. Attenuation of *nodal1* expression compared to unaffected expression of *dand5* in SMIFH2 treated embryos may be explained by suppression of *nodal1* self-enhancement [43] by endodermal covering. An open GRP combined with a strong expression of *tekt2*, a marker of cilia formation at late gastrula in both control and treated embryos indicate that the covering of GRP in treated embryos at neurula stage is due to a premature closure of the GRP rather than by initially abnormal morphogenesis of GRP. Further studies should determine detailed mechanism of this abnormal morphogenesis.

## Supporting information

S1 Fig Embryo survival after exposure to various concentrations of formin inhibitor SMIFH2. Developmental series of DMSO controls and embryos treated with 50 μM of SMIFH2 at the beginning of cleavage (A) or at the start of gastrulation (B). Note 100% lethality for 50μM SMIFH2. Number of embryos treated with 50 μM is 88 for A and 124 for B.
(JPG)

S2 Fig Expression of endodermal marker *sox17* and *tekt2*, a marker of cells forming motile cilia in GRP of embryos treated with DMSO and SMIFH2 from stage 10, fixated at late gastrulation (A and B) and at stage 18 (C and D).
(JPG)

S3 Fig Compilation of *nodal1* (A) and *dand5* (B) expression at stage 18 in embryos treated at gastrula-neurula stages with DMSO and 10 μM SMIFH2.
(JPG)

S4 Fig Representative histological sections through GRP of embryos at stage 18 treated with 10 μM SMIFH2 at stages 10–18, demonstrating position of *nodal1* and *dand5* expressing cells. Arrows indicate areas of superficial *nodal1* and *dand5-*positilklllve cells.
(JPG)

S5 Fig Distribution of actin filaments and cilia in the midline of stage 18 gastrocoel roof plate of embryos treated during gastrula-neurula stages with DMSO (A) or 10 μM SMIFH2 (B) as shown by IF staining using phalloidin (magenta) and antibody against acetylated tubulin (green). Note a narrow midline area bearing long polarized monocilia bordered by wide cells (B). Insets: overview of corresponding regions in SEM.
(JPG)

S6 Fig SEM of GRP of DMSO control embryo and of embryo treated during gastrula-neurula stages with 10 μM SMIFH2 with moderately covered GRP. Arrows–examples of cilia, asterisks–examples of cells devoid of cilia.
(JPG)

S7 Fig Formin inhibition at cleavage stages has no effect on morphology and molecular anatomy of GRP in embryos treated with DMSO and 10 μM SMIFH2. (A) Representative scanning electron microscopy images of GRP. Dorsal explants of stage 18 neurula embryos after formin inhibition at stages 2–6.5. Red color indicates notochord and hypochord, violet–somitic mesoderm, yellow–endoderm. (B-D) Representative images of molecular patterning in the GRP. Dorsal explants of stage 18 neurula embryos after formin inhibition at stages 2–6.5. *In situ* hybridisation was performed with probes specific for *nodal1* (B), *sox17* (C) and *tekt2* (D).
(JPG)

S8 Fig Molecular left-right patterning and GRP morphology after exposure to formin agonist IMM-01. (A) Formin agonist IMM-01 reduced the proportion of left-sided *nodal1* expression at 100 μМ concentration in the embryos treated during cleavage (p-value = 0,0000007118) and during gastrulation and neurulation (p-value = 0,000002126, two-proportions z-test with Bonferroni correction). (B) Formin agonist IMM-01 reduced the proportion of left-sided *pitx2* expression at 100 μМ concentration in the embryos treated during gastrulation and neurulation (p-value = 0,005544, two-proportions z-test with Bonferroni correction). Numbers at the base of columns represent number of analyzed embryos. (C-E) GRP morphology of stage 18 embryos after exposure to 100 μМ formin agonist IMM-01 during gastrulation and neurulation. (C) Cilia polarization, cilia length and percentage of ciliated cells (ciliation rate) in embryos treated with DMSO and 100 μM IMM-01. Formin agonist IMM-01 reduced the average length of cilia at 100 μМ concentration in the embryos treated during gastrulation and neurulation (p-value = 0,001145, t-test with Bonferroni correction). Measurements were performed in notochordal GRP of 11 and 13 embryos respectively (number of analysed cells = 303 and 342 respectively). The apparent decrease of number of ciliated cells is non-significant (p-value = 0,146, t-test with Bonferroni correction). (D) Representative scanning electron microscopy image of GRP. (E) Histological section through the GRP. Blue color indicates ectoderm, red–notochord and hypochord, violet–somitic mesoderm, yellow–endoderm. (F) Distribution of actin filaments and cilia in the midline of stage 18 gastrocoel roof plate as shown by IF staining using phalloidin (magenta) and antibody against acetylated tubulin (green).
(JPG)

## References

[1] Blum M, Feistel K, Thumberger T, Schweickert A. The evolution and conservation of left-right patterning mechanisms. Development. 2014 Apr;141(8):1603–13. doi: 10.1242/dev.100560 . Epub 2014/04/10. eng. 24715452

[2] Blum M, Beyer T, Weber T, Vick P, Andre P, Bitzer E, et al. Xenopus, an ideal model system to study vertebrate left-right asymmetry. Developmental dynamics: an official publication of the American Association of Anatomists. 2009 Jun;238(6):1215–25. doi: 10.1002/dvdy.21855 . Epub 2009/02/12. eng. 19208433

[3] Blum M, Andre P, Muders K, Schweickert A, Fischer A, Bitzer E, et al. Ciliation and gene expression distinguish between node and posterior notochord in the mammalian embryo. Differentiation; research in biological diversity. 2007 Feb;75(2):133–46. doi: 10.1111/j.1432-0436.2006.00124.x . Epub 2007/02/24. eng. 17316383

[4] Essner JJ, Vogan KJ, Wagner MK, Tabin CJ, Yost HJ, Brueckner M. Conserved function for embryonic nodal cilia. Nature. 2002 Jul 4;418(6893):37–8. doi: 10.1038/418037a . Epub 2002/07/05. eng. 12097899

[5] Nonaka S, Shiratori H, Saijoh Y, Hamada H. Determination of left-right patterning of the mouse embryo by artificial nodal flow. Nature. 2002 Jul 4;418(6893):96–9. doi: 10.1038/nature00849 . 12097914

[6] Tisler M, Wetzel F, Mantino S, Kremnyov S, Thumberger T, Schweickert A, et al. Cilia are required for asymmetric nodal induction in the sea urchin embryo. BMC developmental biology. 2016 Aug 23;16(1):28. doi: 10.1186/s12861-016-0128-7 . Epub 2016/08/25. eng. 27553781 PMC4994401

[7] Schweickert A, Vick P, Getwan M, Weber T, Schneider I, Eberhardt M, et al. The nodal inhibitor Coco is a critical target of leftward flow in Xenopus. Curr Biol. 2010 Apr 27;20(8):738–43. doi: 10.1016/j.cub.2010.02.061 . Epub 2010/04/13. eng. 20381352

[8] Yoshiba S, Hamada H. Roles of cilia, fluid flow, and Ca signaling in breaking of left-right symmetry. Trends in genetics: TIG. 2013 Sep 30. doi: 10.1016/j.tig.2013.09.001 . Epub 2013/10/05. Eng. 24091059

[9] Maerker M, Getwan M, Dowdle ME, McSheene JC, Gonzalez V, Pelliccia JL, et al. Bicc1 and Dicer regulate left-right patterning through post-transcriptional control of the Nodal inhibitor Dand5. Nature communications. 2021 Sep 16;12(1):5482. doi: 10.1038/s41467-021-25464-z . 34531379 PMC8446035

[10] Manner J. Does an equivalent of the "ventral node" exist in chick embryos? A scanning electron microscopic study. Anat Embryol (Berl). 2001 Jun;203(6):481–90. doi: 10.1007/s004290100183 . 11453165

[11] Tsikolia N, Schroder S, Schwartz P, Viebahn C. Paraxial left-sided nodal expression and the start of left-right patterning in the early chick embryo. Differentiation; research in biological diversity. 2012 Dec;84(5):380–91. doi: 10.1016/j.diff.2012.09.001 . Epub 2012/11/13. eng. 23142734

[12] Gros J, Feistel K, Viebahn C, Blum M, Tabin CJ. Cell movements at Hensen’s node establish left/right asymmetric gene expression in the chick. Science. 2009 May 15;324(5929):941–4. doi: 10.1126/science.1172478 . 19359542 PMC2993078

[13] Schroder SS, Tsikolia N, Weizbauer A, Hue I, Viebahn C. Paraxial Nodal Expression Reveals a Novel Conserved Structure of the Left-Right Organizer in Four Mammalian Species. Cells, tissues, organs. 2016;201(2):77–87. doi: 10.1159/000440951 . 26741372

[14] Kajikawa E, Horo U, Ide T, Mizuno K, Minegishi K, Hara Y, et al. Nodal paralogues underlie distinct mechanisms for visceral left-right asymmetry in reptiles and mammals. Nature ecology & evolution. 2020 Feb;4(2):261–9. doi: 10.1038/s41559-019-1072-2 . 31907383

[15] Adams DS, Robinson KR, Fukumoto T, Yuan S, Albertson RC, Yelick P, et al. Early, H+-V-ATPase-dependent proton flux is necessary for consistent left-right patterning of non-mammalian vertebrates. Development. 2006 May;133(9):1657–71. doi: 10.1242/dev.02341 . 16554361 PMC3136117

[16] Vandenberg LN, Levin M. A unified model for left-right asymmetry? Comparison and synthesis of molecular models of embryonic laterality. Developmental biology. 2013 Jul 1;379(1):1–15. doi: 10.1016/j.ydbio.2013.03.021 . Epub 2013/04/16. eng. 23583583 PMC3698617

[17] Levin M, Palmer AR. Left-right patterning from the inside out: widespread evidence for intracellular control. BioEssays: news and reviews in molecular, cellular and developmental biology. 2007 Mar;29(3):271–87. doi: 10.1002/bies.20545 . 17295291

[18] Fukumoto T, Blakely R, Levin M. Serotonin transporter function is an early step in left-right patterning in chick and frog embryos. Developmental neuroscience. 2005;27(6):349–63. doi: 10.1159/000088451 . 16280633

[19] Walentek P, Beyer T, Thumberger T, Schweickert A, Blum M. ATP4a is required for Wnt-dependent Foxj1 expression and leftward flow in Xenopus left-right development. Cell Rep. 2012 May 31;1(5):516–27. doi: 10.1016/j.celrep.2012.03.005 . Epub 2012/07/27. eng. 22832275

[20] Beyer T, Danilchik M, Thumberger T, Vick P, Tisler M, Schneider I, et al. Serotonin signaling is required for Wnt-dependent GRP specification and leftward flow in Xenopus. Curr Biol. 2012 Jan 10;22(1):33–9. doi: 10.1016/j.cub.2011.11.027 . Epub 2011/12/20. eng. 22177902

[21] Vandenberg LN, Levin M. Polarity proteins are required for left-right axis orientation and twin-twin instruction. Genesis. 2012 Mar;50(3):219–34. doi: 10.1002/dvg.20825 . Epub 2011/11/17. eng. 22086838 PMC3294047

[22] Blum M, Schweickert A, Vick P, Wright CV, Danilchik MV. Symmetry breakage in the vertebrate embryo: when does it happen and how does it work? Developmental biology. 2014 Sep 1;393(1):109–23. doi: 10.1016/j.ydbio.2014.06.014 . Epub 2014/06/28. eng. 24972089 PMC4481729

[23] Inaki M, Sasamura T, Matsuno K. Cell Chirality Drives Left-Right Asymmetric Morphogenesis. Frontiers in cell and developmental biology. 2018;6:34. doi: 10.3389/fcell.2018.00034 . 29666795 PMC5891590

[24] Dimonte A, Adamatzky A, Erokhin V, Levin M. On chirality of slime mould. Bio Systems. 2016 Feb; 140:23–7. doi: 10.1016/j.biosystems.2015.12.008 . Epub 2016/01/10. eng. 26747637

[25] Naganathan SR, Furthauer S, Nishikawa M, Julicher F, Grill SW. Active torque generation by the actomyosin cell cortex drives left-right symmetry breaking. eLife. 2014;3:e04165. doi: 10.7554/eLife.04165 . Epub 2014/12/18. eng. 25517077 PMC4269833

[26] Abe M, Kuroda R. The development of CRISPR for a mollusc establishes the formin Lsdia1 as the long-sought gene for snail dextral/sinistral coiling. Development. 2019 May 14;146(9). doi: 10.1242/dev.175976 . 31088796

[27] Davison A, McDowell GS, Holden JM, Johnson HF, Koutsovoulos GD, Liu MM, et al. Formin Is Associated with Left-Right Asymmetry in the Pond Snail and the Frog. Curr Biol. 2016 Mar 7;26(5):654–60. doi: 10.1016/j.cub.2015.12.071 . Pubmed Central PMCID: 4791482. Epub 2016/03/01. eng. 26923788 PMC4791482

[28] Kay BK, Peng HB. Xenopus laevis: practical uses in cell and molecular biology.: Academic Press; 1992.

[29] Nieuwkoop PD, Faber J. Normal table of Xenopus laevis (Daudin): a systematical and chronological survey of the development from the fertilized egg till the end of metamorphosis. New York: Garland Pub.; 1994. 252 p., 10 leaves of plates p.

[30] Shook DR, Majer C, Keller R. Pattern and morphogenesis of presumptive superficial mesoderm in two closely related species, Xenopus laevis and Xenopus tropicalis. Developmental biology. 2004 Jun 1;270(1):163–85. doi: 10.1016/j.ydbio.2004.02.021 . 15136148

[31] Stubbs JL, Oishi I, Izpisua Belmonte JC, Kintner C. The forkhead protein Foxj1 specifies node-like cilia in Xenopus and zebrafish embryos. Nat Genet. 2008 Dec;40(12):1454–60. doi: 10.1038/ng.267 . 19011629 PMC4648715

[32] Harland RM. In situ hybridization: an improved whole-mount method for Xenopus embryos. Methods Cell Biol. 1991;36:685–95. doi: 10.1016/s0091-679x(08)60307-6 . 1811161

[33] Rizvi SA, Neidt EM, Cui J, Feiger Z, Skau CT, Gardel ML, et al. Identification and characterization of a small molecule inhibitor of formin-mediated actin assembly. Chemistry & biology. 2009 Nov 25;16(11):1158–68. doi: 10.1016/j.chembiol.2009.10.006 . 19942139 PMC2784894

[34] Isogai T, van der Kammen R, Innocenti M. SMIFH2 has effects on Formins and p53 that perturb the cell cytoskeleton. Scientific reports. 2015 Apr 30;5:9802. doi: 10.1038/srep09802 . 25925024 PMC5386218

[35] Tisler M, Thumberger T, Schneider I, Schweickert A, Blum M. Leftward flow determines laterality in conjoined twins. Current Biology. 2017 27(4): 543–548. doi: 10.1016/j.cub.2016.12.049 . 28190730

[36] Nishimura Y, Shi S, Zhang F, Liu R, Takagi Y, Bershadsky AD, et al. The formin inhibitor SMIFH2 inhibits members of the myosin superfamily. Journal of cell science. 2021 Apr 15;134(8). doi: 10.1242/jcs.253708 . 33589498 PMC8121067

[37] Moody SA. Fates of the blastomeres of the 32-cell-stage Xenopus embryo. Developmental biology. 1987 Aug;122(2):300–19. doi: 10.1016/0012-1606(87)90296-x . 3596014

[38] Moody SA. Fates of the blastomeres of the 16-cell stage Xenopus embryo. Developmental biology. 1987 Feb;119(2):560–78. doi: 10.1016/0012-1606(87)90059-5 . 3803718

[39] Tingler M., et al., Symmetry breakage in the frog Xenopus: role of Rab11 and the ventral-right blastomere. Genesis, 2014. Tingler M., et al., Symmetry breakage in the frog Xenopus: role of Rab11 and the ventral-right blastomere. Genesis, 2014. 52(6): p. 588–99. 52(6): p. 588–99. doi: 10.1002/dvg.22766 24585437

[40] Lash LL, Wallar BJ, Turner JD, Vroegop SM, Kilkuskie RE, et al. Small-molecule intramimics of formin autoinhibition: a new strategy to target the cytoskeletal remodeling machinery in cancer cells. Cancer research. 2013; 73(22), 6793–6803. doi: 10.1158/0008-5472.CAN-13-1593 . 24242070

[41] Shinohara K, Kawasumi A, Takamatsu A, Yoshiba S, Botilde Y, Motoyama N, et al. Two rotating cilia in the node cavity are sufficient to break left-right symmetry in the mouse embryo. Nature communications. 2012; 3:622. doi: 10.1038/ncomms1624 . Epub 2012/01/12. eng. 22233632

[42] Schneider I, Kreis J, Schweickert A, Blum M, Vick P. A dual function of FGF signaling in Xenopus left-right axis formation. Development. 2019 May 10;146(9). doi: 10.1242/dev.173575 . 31036544

[43] Nakamura T, Mine N, Nakaguchi E, Mochizuki A, Yamamoto M, Yashiro K, et al. Generation of robust left-right asymmetry in the mouse embryo requires a self-enhancement and lateral-inhibition system. Dev Cell. 2006 11(4):495–504. doi: 10.1016/j.devcel.2006.08.002 . 17011489

