# Supplementary figures and images for: Abnormal left-right organizer and laterality defects in Xenopus embryos after formin inhibitor SMIFH2 treatment
Source: PLoS One. 2022 Nov 7;17(11):e0275164. doi: 10.1371/journal.pone.0275164 (PMC9639825; doi:10.1371/journal.pone.0275164)

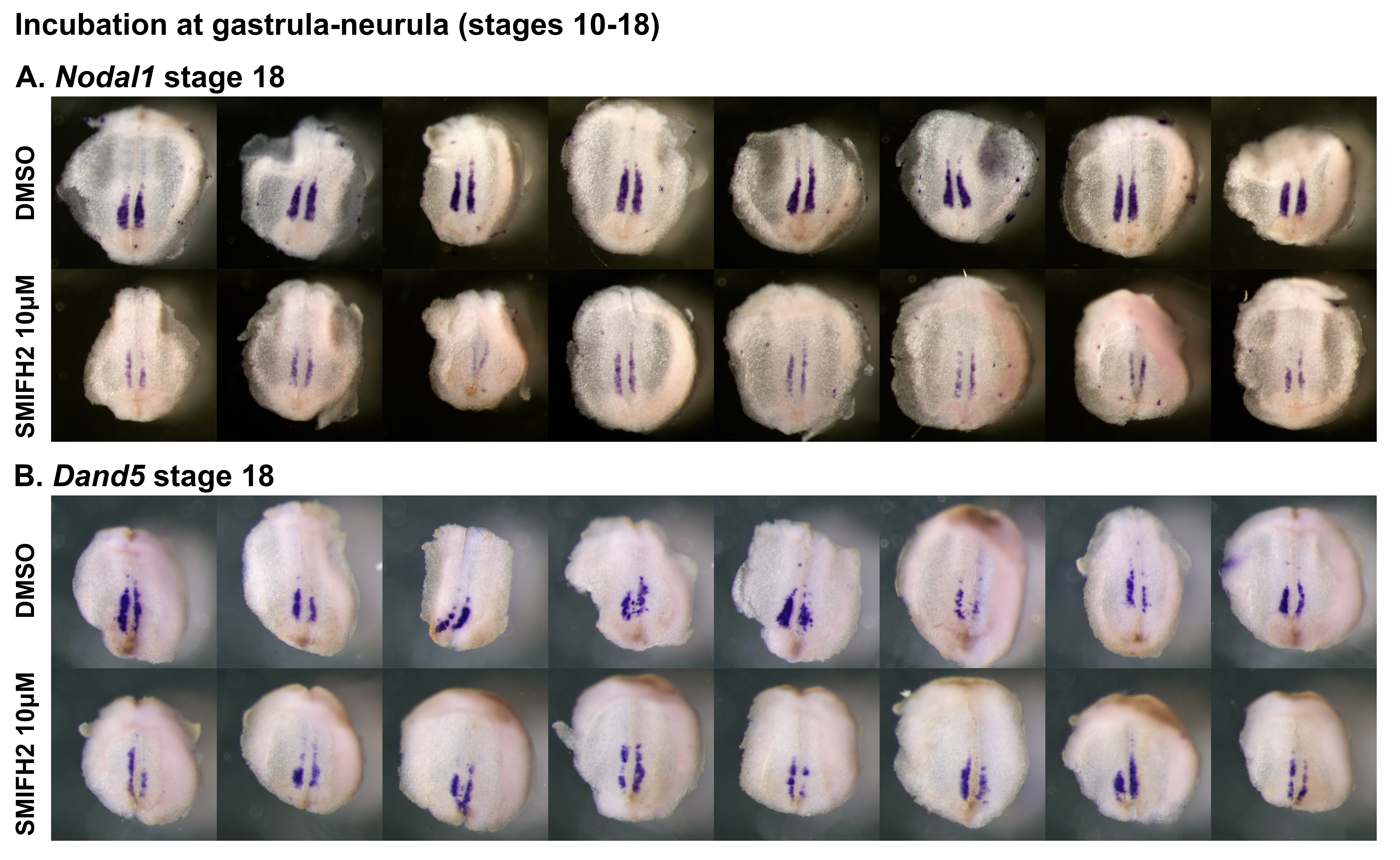

Supplement: S3 Fig — Compilation of nodal1 (A) and dand5 (B) expression at stage 18 in embryos treated at gastrula-neurula stages with DMSO and 10 μM SMIFH2. (JPG) [file pone.0275164.s003.jpg]

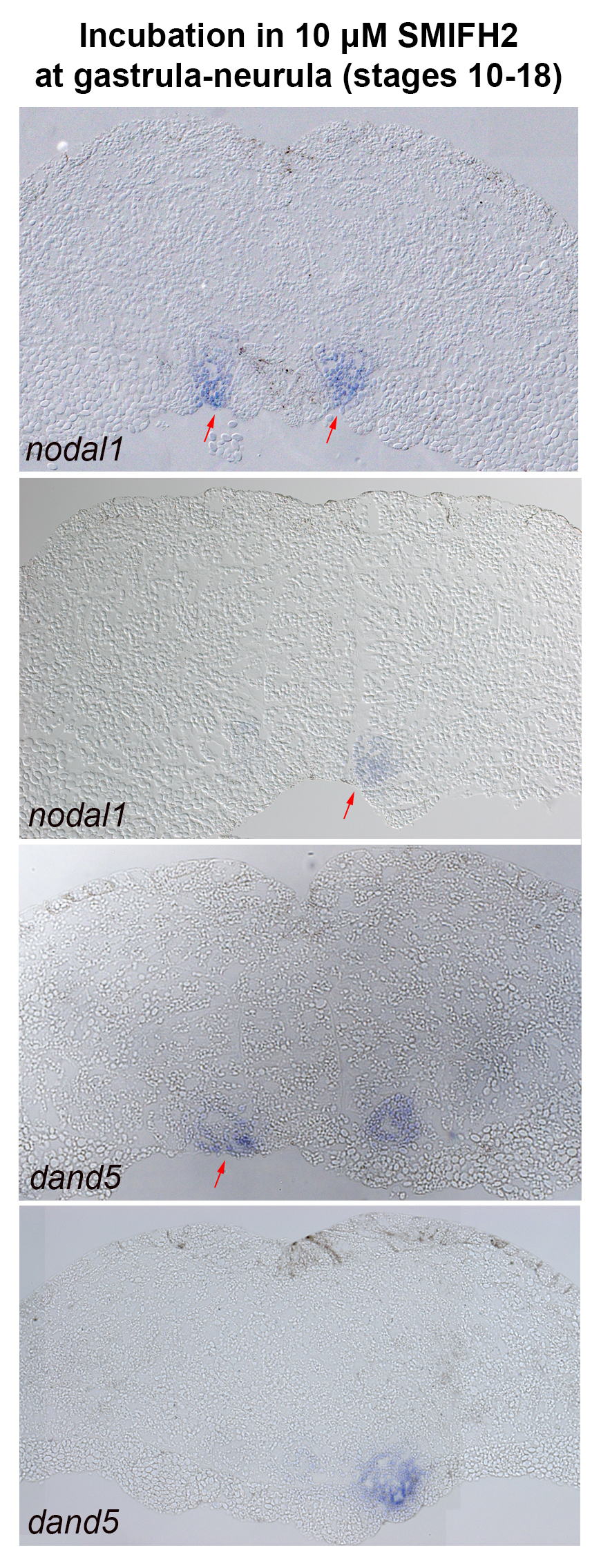

Supplement: S4 Fig — Arrows indicate areas of superficial nodal1 and dand5-positilklllve cells. (JPG) [file pone.0275164.s004.jpg]

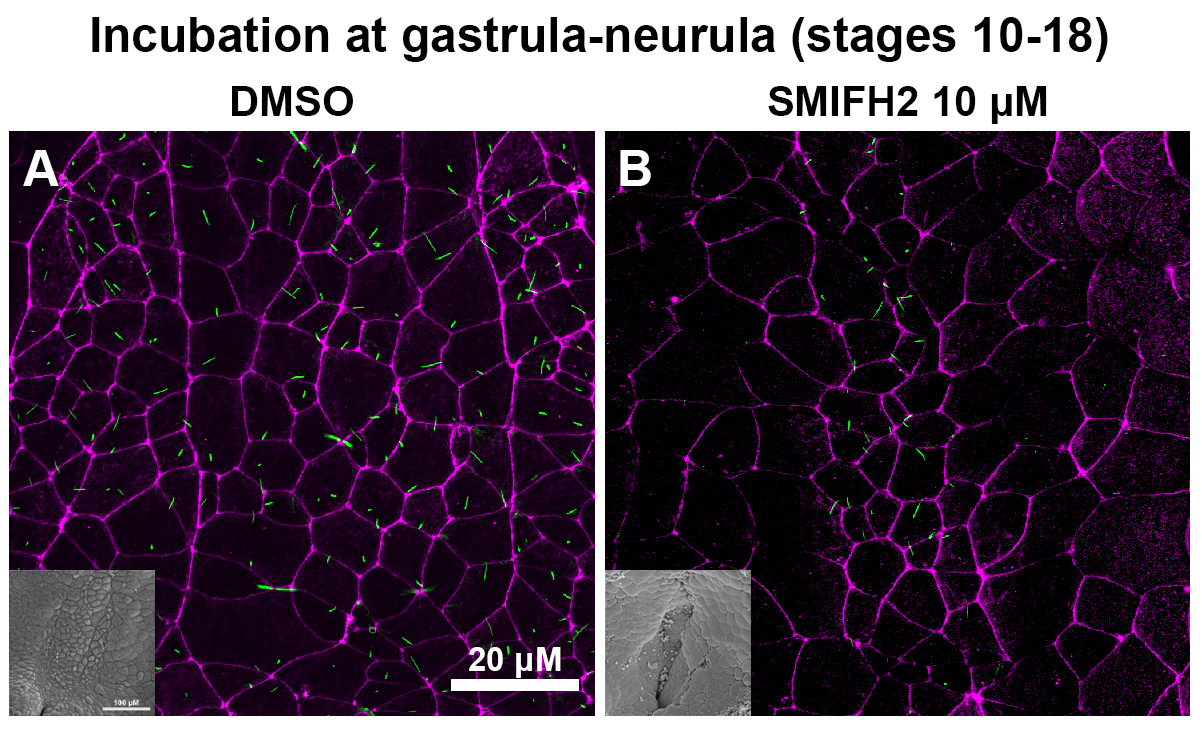

Supplement: S5 Fig — Distribution of actin filaments and cilia in the midline of stage 18 gastrocoel roof plate of embryos treated during gastrula-neurula stages with DMSO (A) or 10 μM SMIFH2 (B) as shown by IF staining using phalloidin (magenta) and antibody against acetylated tubulin (green). Note a narrow midline area bearing long polarized monocilia bordered by wide cells (B). Insets: overview of corresponding regions in SEM. (JPG) [file pone.0275164.s005.jpg]

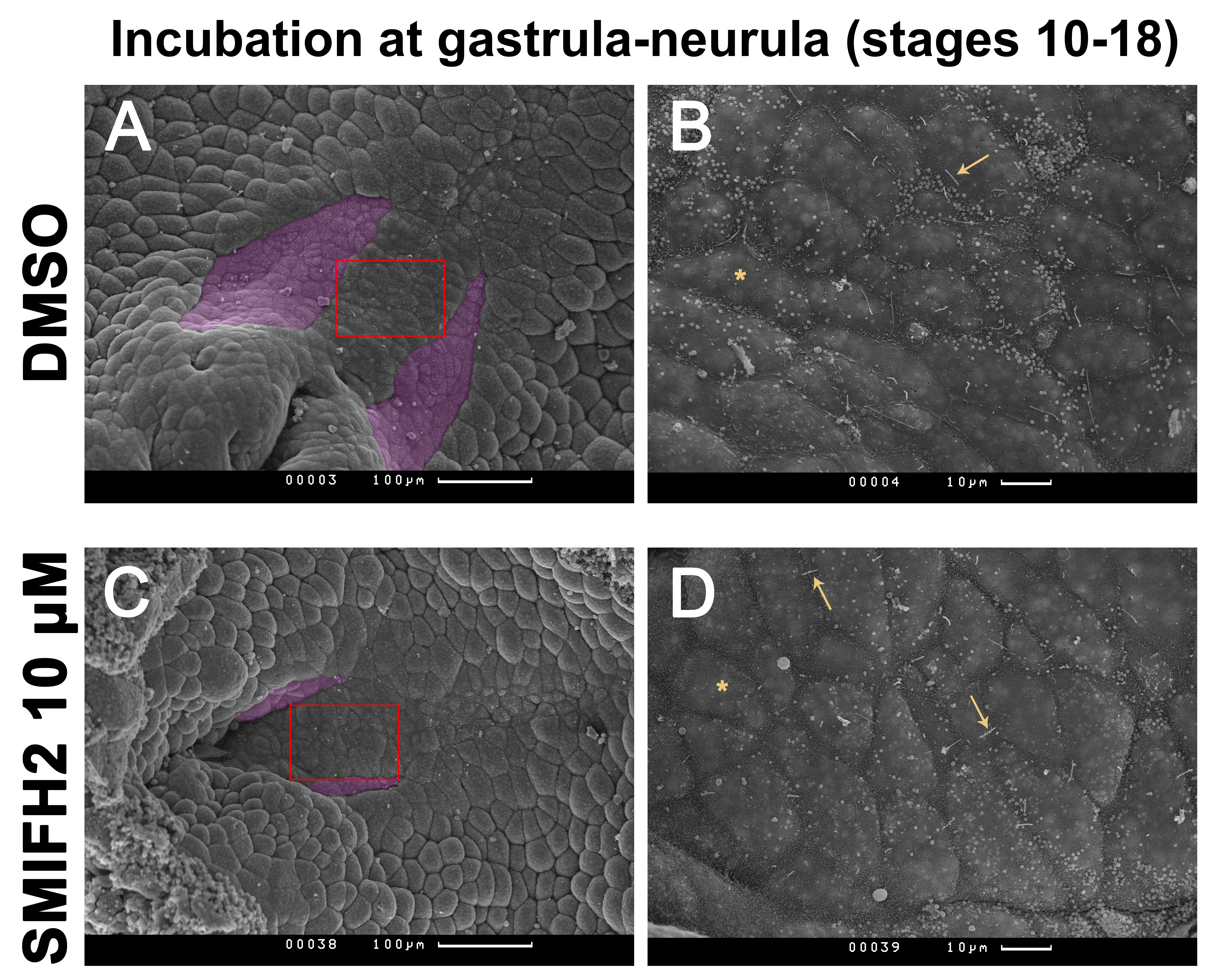

Supplement: S6 Fig — Arrows–examples of cilia, asterisks–examples of cells devoid of cilia. (JPG) [file pone.0275164.s006.jpg]

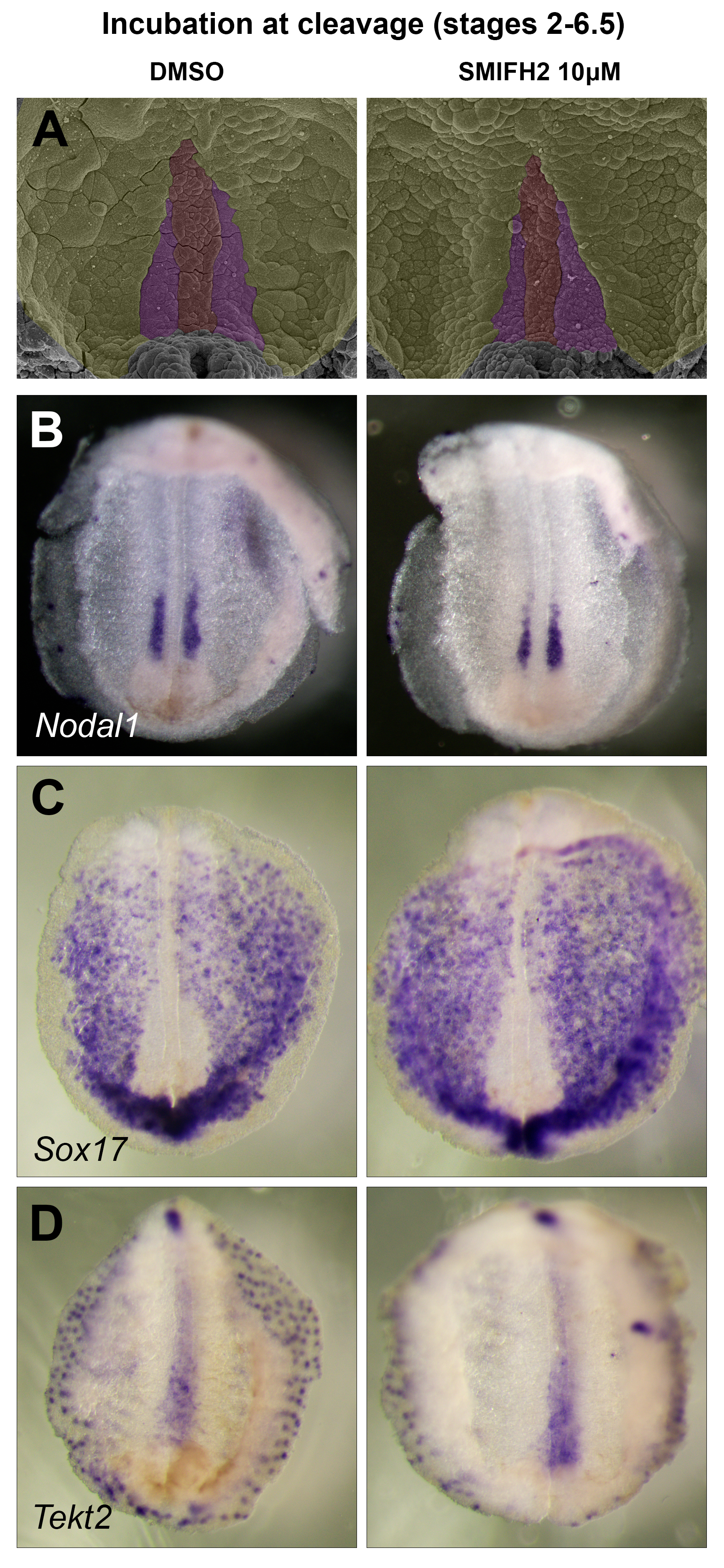

Supplement: S7 Fig — (A) Representative scanning electron microscopy images of GRP. Dorsal explants of stage 18 neurula embryos after formin inhibition at stages 2–6.5. Red color indicates notochord and hypochord, violet–somitic mesoderm, yellow–endoderm. (B-D) Representative images of molecular patterning in the GRP. Dorsal explants of stage 18 neurula embryos after formin inhibition at stages 2–6.5. In situ hybridisation was performed with probes specific for nodal1 (B), sox17 (C) and tekt2 (D). (JPG) [file pone.0275164.s007.jpg]

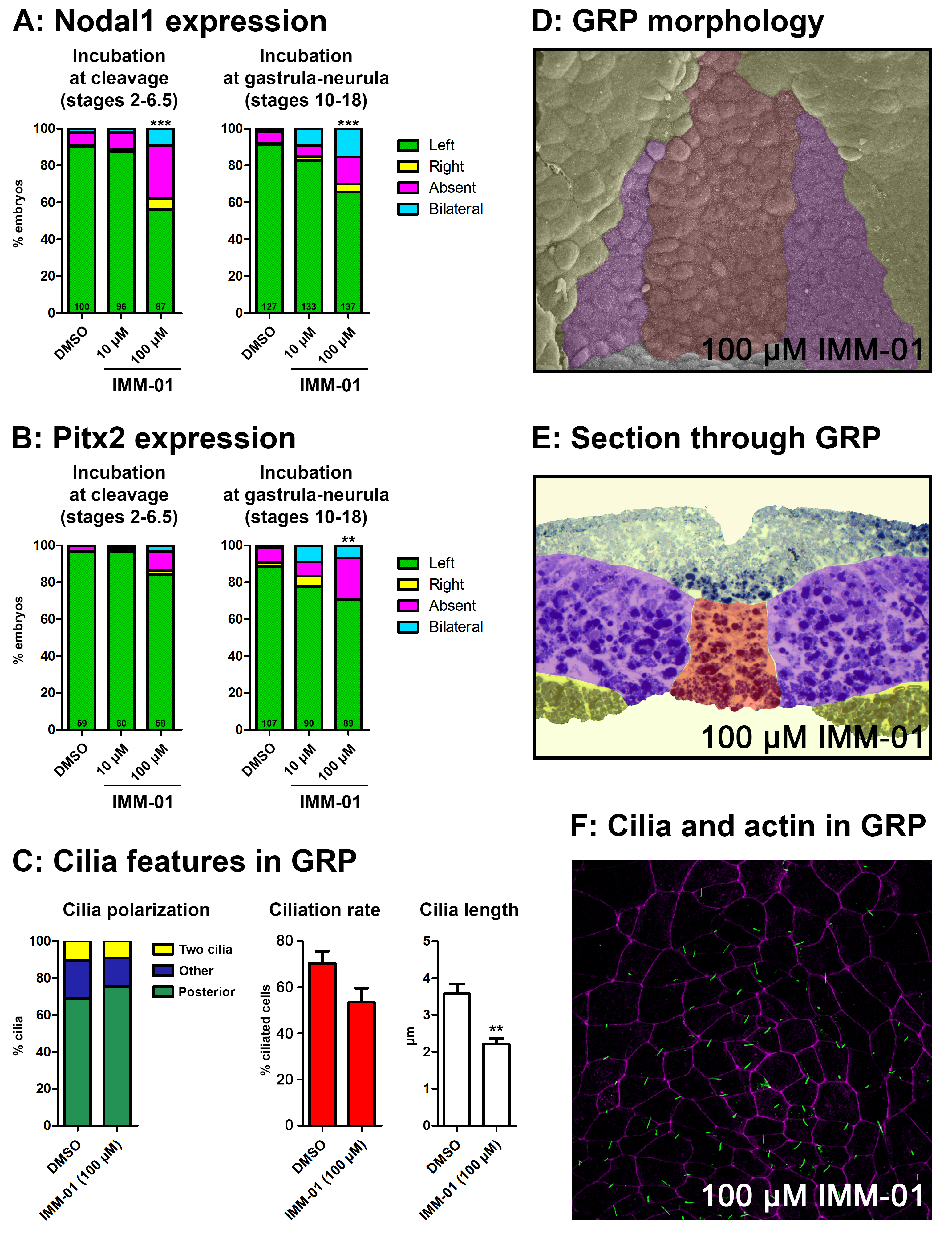

Supplement: S8 Fig — (A) Formin agonist IMM-01 reduced the proportion of left-sided nodal1 expression at 100 μМ concentration in the embryos treated during cleavage (p-value = 0,0000007118) and during gastrulation and neurulation (p-value = 0,000002126, two-proportions z-test with Bonferroni correction). (B) Formin agonist IMM-01 reduced the proportion of left-sided pitx2 expression at 100 μМ concentration in the embryos treated during gastrulation and neurulation (p-value = 0,005544, two-proportions z-test with Bonferroni correction). Numbers at the base of columns represent number of analyzed embryos. (C-E) GRP morphology of stage 18 embryos after exposure to 100 μМ formin agonist IMM-01 during gastrulation and neurulation. (C) Cilia polarization, cilia length and percentage of ciliated cells (ciliation rate) in embryos treated with DMSO and 100 μM IMM-01. Formin agonist IMM-01 reduced the average length of cilia at 100 μМ concentration in the embryos treated during gastrulation and neurulation (p-value = 0,001145, t-test with Bonferroni correction). Measurements were performed in notochordal GRP of 11 and 13 embryos respectively (number of analysed cells = 303 and 342 respectively). The apparent decrease of number of ciliated cells is non-significant (p-value = 0,146, t-test with Bonferroni correction). (D) Representative scanning electron microscopy image of GRP. (E) Histological section through the GRP. Blue color indicates ectoderm, red–notochord and hypochord, violet–somitic mesoderm, yellow–endoderm. (F) Distribution of actin filaments and cilia in the midline of stage 18 gastrocoel roof plate as shown by IF staining using phalloidin (magenta) and antibody against acetylated tubulin (green). (JPG) [file pone.0275164.s008.jpg]
